# Anti-atherogenic properties of vitamin E, aspirin, and their combination

**DOI:** 10.1371/journal.pone.0206315

**Published:** 2018-10-25

**Authors:** Sheau C. Chai, Elizabeth M. Foley, Bahram H. Arjmandi

**Affiliations:** 1 Department of Behavioral Health and Nutrition, College of Health Sciences, University of Delaware, Newark, DE, United State of America; 2 Department of Nutrition, Food and Exercise Sciences, College of Human Sciences, Florida State University, Tallahassee, FL, United State of America; 3 Center for Advancing Exercise and Nutrition Research on Aging (CAENRA), College of Human Sciences, Florida State University, Tallahassee, FL, United State of America; Michigan State University, UNITED STATES

## Abstract

The present study was designed to assess the extent to which vitamin E and aspirin individually or in combination prevent and/or reverse bone loss and atherosclerotic lesion formation in orchidectomized aged rats. Forty-nine 12-month old male Sprague-Dawley rats were either sham-operated (Sham, one group) or orchidectomized (Orx, four groups) and fed a control diet for 120 days to establish bone loss and atherosclerotic lesions. Thereafter, rats were assigned to the various treatment groups (n = 9 to 10 per group): 1) Sham and 2) Orx groups received AIN93M, containing 75 IU vitamin E and served as control, and the other three Orx groups received either 3) 500 IU vitamin E, 4) 500 mg aspirin, or 5) 500 IU vitamin E + 500 mg aspirin per kg diet for 90 days. After 90 days of treatment, rats were sacrificed, necropsied, and tissues were collected for analyses. Results show that 500 IU vitamin E was able to reduce the development of atherosclerosis lesion formation and aortic streak area compared to Orx control. More importantly, 500 mg aspirin completely reversed the fatty streak area and made the atherosclerotic lesions disappear. Vitamin E and aspirin were not able to reverse bone loss as shown by whole body, lumbar and femoral bone mineral content and bone mineral density due to gonadal hormone deficiency. Instead, 500 mg aspirin somewhat increased the trabecular separation while decreased trabecular thickness compared to Orx control. Our findings suggest that both, vitamin E and aspirin exert anti-atherogenic effects and aspirin is more effective than vitamin E in preventing atherosclerosis lesions in Orx rats.

## Introduction

Cardiovascular disease and osteoporosis are often associated with aging. Atherosclerosis causes one-third of all deaths in the United States [1], and is characterized by fatty lesion development along the walls of the coronary arteries which reduces blood flow [2]. Decline of testosterone levels in men has been suggested to influence lipoprotein metabolism including higher total cholesterol (TC), triglycerides (TG) and low-density lipoprotein cholesterol (LDL), all of which are associated with increased risk of atherosclerosis [3]. Oxidative stress is a proposed mechanism by which atherosclerosis develops, with oxygen derived free radicals causing an increase in inflammation and inflammatory mediators [4]. Reactive oxygen species (ROS) can also damage the endothelium and lead to atherogenesis, which is the development of fatty deposits known as plaques [5]. In addition to directly damaging cell membranes, ROS peroxidizes lipid components in the form of LDL [4]; these newly formed plaques can then rupture, creating a cardiovascular event known as a myocardial infarction (MI) which can result in death.

Vitamin E supplementation is a popular way to prevent cardiovascular disease (CVD) [6]. Indeed, studies have shown that vitamin E can be anti-inflammatory by inhibiting cyclooxygenase (COX)-2 [7], as well as functioning as a potent antioxidant [8]. Theoretically, anti-oxidative properties would work to decelerate atherosclerotic lesion development; however, many studies have found that its supplementation has had little to no benefit on CVD or its outcomes [9, 10]. The effect of supplemental doses of vitamin E on atherosclerosis has yielded conflicting results. For instance, several studies [11–14] reported a lack of evidence supporting the use of antioxidant supplementation in preventing atherosclerotic lesions. This discrepancy in findings between interventional and observational studies is most likely due to the fact that the majority of these clinical trials recruited patients with pre-existing cardiovascular events or at risk of CVD. In addition, the type, dosage, and combinations of antioxidants used varied between trials resulting in inconsistencies. Conversely, one of our previous studies has shown that vitamin E dose-dependently reduces aortic fatty lesion formation in orchidectomized (Orx) rats [15], likely due to its anti-oxidative effects rather than decreasing inflammation.

Vitamin E is not the only popular supplement used to reduce the risk of a cardiovascular event. Many Americans use aspirin as a way to prevent heart disease; indeed, a survey regarding aspirin use among adults over age 40 reported that 41% of those surveyed expressed regular aspirin use for CVD prevention [16]. Aspirin is a well-known anti-inflammatory agent with medicinal uses that date back to Hippocrates [17]. Much like vitamin E, aspirin works to inhibit COX; this inhibition also works to inhibit prostaglandin (PG) production, which cause swelling and inflammation [18]. Interestingly, observational studies regarding aspirin usage have yielded conflicting results in efficacy. A recent study by Bavry et al. [19] found that aspirin was only slightly beneficial for atherosclerotic patients that had experienced a prior ischemic event, but was not beneficial for patients that had never experienced an ischemic event. Another study found that nonfatal MI risk was reduced by 22%, and MI benefit is seen to be most prominent in individuals aged 65 and older [20]. Since vitamin E and aspirin are taken by a large number of American for preventing CVD, the effectiveness of combined aspirin and vitamin E treatment has hardly been studied.

Atherosclerosis develops as a result of a chronic inflammatory response and age [21], much like the pathology of osteoporosis [22]. Though multiple factors play a role in the development of osteoporosis, low grade inflammation has been postulated to be a main contributor [23, 24]. Because PGs have been implicated in CVD [25] and are also involved in the bone remodeling process, often resulting in bone resorption exceeding that of bone formation, it is natural to theorize that the inhibition of these products would decrease incidence of osteoporosis, as well as incidence of atherosclerosis.

Aspirin may also be effective in reducing bone loss by preventing the formation of osteoclasts via inhibition of nuclear factor-kB, which would ultimately stimulate the formation of osteoblasts [26]. A study by Yamaza et al. [27] supplementing aspirin in ovariectomized mice proved to be successful in preventing trabecular and cortical density loss. Aspirin has been found to improve bone structure without effecting the rate of bone formation [28], decrease receptor activator of nuclear factor kappa-B ligand expression in the bone [29], and to increase bone mineral density (BMD) as well as improve biomechanical properties of bone [30].

Osteoporosis in men is widely understudied, despite the fact that one third of all hip fractures worldwide occur in men [31] and that mortality in men during the first year after hip fracture is 51% higher than in women [32]. Additionally, the effect of aspirin usage on bone health has not been widely studied, though some studies have found it to be associated with higher BMD in both men and women [33]. Because aspirin supplementation is so widely suggested to decrease the risk of CVD, it is important to investigate the effects of aspirin on bone health, both to ensure that one disease is not being traded in for the other, and to detect a potential positive benefit on bone health. Therefore, we conducted a study to investigate the extent to which vitamin E and aspirin individually or in combination prevent and/or reverse bone loss and atherosclerotic lesion formation in orchidectomized aged rats.

## Materials and methods

### Animals and diet

Forty-nine, 12-month old male Sprague-Dawley rats (Harlan, Indianapolis, IN) were housed in an environmentally controlled laboratory. All of the experimental procedures involving the use of animals were approved by the Animal Care and Use Committee at Oklahoma State University. After three days of acclimation, rats were either sham-operated (Sham; 1 group) or orchidectomized (Orx; 4 groups) with 9–10 rats in each group. After surgery, all rats were fed an AIN-93M casein-based control diet (Harlan, Madison, WI) for 120 days to establish bone loss. One hundred-twenty days from the date of surgery, the Sham group and one Orx group continued to receive control diet. The other 3 groups received supplemental dose of vitamin E in the form of DL-α-tocopherol acetate as follows: Orx + 500 IU vitamin E, Orx + 500 mg aspirin, and Orx + 500 IU vitamin E + 500 mg aspirin per kg diet. Aspirin was added to the AIN-93M semi-purified powder diet according to the methods shown by previous studies [34–36]. This rational was used to mimic the human use of aspirin. Rats were pair-fed to the mean food intake of the group that consumed the least. Deionized water access was unrestricted. Food intake was determined every three days and body weight was determined every week. These rats were fed their respective dietary treatment for 90 days and then sacrificed at the end of the treatment period.

### Animals necropsy and collection of heart, urine, blood and bone samples

At the end of the 90 days, rats were fasted and placed in metabolic cages. Urine was collected for 12 hours. Prior to necropsy, rats were anesthetized with a mixture of ketamine and xylazine at 70 mg and 3 mg/kg body weight, respectively, and then whole body BMD and BMC were measured using dual energy X-ray absorptiometry (DXA, model QDR-4500A Elite, Hologic Waltham, MA). The animals were bled from their abdominal aortas for blood sample collection. Heart were removed immediately, rinsed with ice-cold saline to remove blood, and weighted. Urine and serum were centrifuged at 1500 x g for 20 minutes at 4°C, aliquotted and stored at -20°C until analysis. Heart samples were kept in 10% formalin in refrigerator until the evaluation of atherosclerotic lesions. Femurs and vertebrae were collected, cleaned of adhering tissues and stored at -20°C until analysis.

### Serum total-, HDL-cholesterol, and triglycerides

Serum TC, high-density lipoprotein cholesterol (HDL), and TG levels were measured using kits from Roche Diagnostics (Nutley, NJ). These tests were performed using a Clinical Analyzer (Montclair, NJ) following the manufacturer’s instructions.

### Assessment of atherosclerotic lesions

As described in detail previously [15], atherosclerotic lesions in the aortic arch were evaluated as described by Auger et al. [37] because several studies [38, 39] have shown that this area is especially prone to atherosclerosis with a clear starting point (aortic valves). Briefly, the aorta was cleaned of peripheral fat, sectioned, embedded in tissue freezing medium (O.C.T. compound; Sakura Finetek, Torrance, CA), and frozen at -20°C. Sections 10-μm thick were prepared using a cryostat (Bright Instrument Co., Huntingdon, UK) and thaw-mounted onto glass slides coated with 3% gelatin. Sections were evaluated for fatty streak lesions after staining with Oil red O (Sigma, St. Louis, MO) and counterstaining with Harris' hematoxylin (Sigma). The Oil red O-stained area was analyzed quantitatively using a computer-assisted morphometry system and expressed as a percentage of the total area surveyed. In addition, the degree of lesion formation was classified as normal (without lesion formation), early atheromatous, atheromatous, or severe atheromatous plaque based on assigning the level of calcification or fibrous tissue formation as judged by the same pathologist in a blinded fashion.

### Serum and urine biomarkers of bone metabolism

Urinary excretion of deoxypyridinoline (Dpd), a specific marker of bone resorption was measured using a competitive enzyme immunoassay in a microtiter stripwell format (Metra DPD EIA Kit, Quidel Corporation, CA) and a microplate reader (ELx808 Ultra Microplate Reader, Bio-Tek, VT). Serum osteocalcein (OC) level as a marker of bone formation was assessed by two-site immunoradiometric assay (Immunotopics, Inc., San Clemente, CA).

### Bone density and content assessments

Whole body was scanned using DXA at three time points; baseline (before surgery), 120 days after surgery, and after 90 days of treatment. At the end of a 90-day treatment period, the spine and femurs were removed, cleaned of adhering tissue and then the region of lumbar vertebrae and right femur were measured using DXA to determine BMD, bone mineral content (BMC), and bone mineral area (BMA) for final evaluation.

### Microcomputed tomography analysis of distal femur

As described in detail previously [40], the microarchitectural trabecular structure of the distal femoral metaphysis was evaluated using micro-computed tomography (MicroCT40, Scanco Medical, Switzerland). The distal femur was scanned from the growth plate in the proximal direction (16 μm/slice). This region included 350 images obtained from each femur using 1024 x 1024 matrix resulting in an isotropic voxel resolution of 22 μm3. An integration time of 70 milliseconds per projection was used, with a rotational step of 0.36 degrees. The volume of interest (VOI) was selected as a region 25 slices away from the growth plate at the distal end of the femur to 125 slices. The trabecular bone morphometric parameters assessed with the micro-CT included the bone volume expressed as a percentage of total volume (BV/TV), trabecular number (Tb.N), trabecular separation (Tb.Sp), and trabecular thickness (Tb.Th). Non-metric parameters included structural model index (SMI), and connectivity density (Conn.D).

### Statistical analysis

Statistical analysis was performed using analysis of variance (ANOVA) with PROC MIXED in SAS Version 9.3 (SAS Institute, Cary, NC). If post hoc analysis showed statistical significance, Fisher's least significant difference test was used to determine and compare the significant differences among the mean of each treatment groups. Data are reported as means and standard error for each of the treatment groups. In all statistical comparisons, differences with *P*<0.05 were considered significant. For data on atherosclerotic lesion, a chi-square test was used to compare the frequency of incidence and progression of atherosclerotic lesion among the treatment groups.

## Results

### Food intake, body weight, and coagulating gland weight

The effects of Orx and the supplemental doses of vitamin E, aspirin, and their combination on food intake, body and coagulating gland weights are presented in Table 1. There were no statistical differences among any of the treatment groups in terms of food intake and body weights. As expected, all rats in Orx groups had lower (*P*<0.0001) coagulating gland weights in comparison with Sham animals confirming the success of Orx.

**Table 1:** Effects of orchidectomy (Orx), vitamin E and aspirin on food intake, body and coagulating gland weights.

	Sham	Orx			
Treatment (per kg diet)	Control	Control	500 IU Vit E	500 mg Aspirin	500 IU Vit E + 500 mg Aspirin
Average food intake (g/day)	16.5±0.3	15.9±0.2	16.1±0.2	16.0±0.2	16.1±0.2
Initial body weight (g)	489±7.9	487±10.5	488±9.7	488±9.4	488±9.4
Final body weight (g)	482±15.7	465±13.4	503±17.7	484±14.2	487±14.5
Coagulating gland (g)	1.36±0.20^a^	0.18±0.0^b^	0.18±0.01^b^	0.16±0.02^b^	0.19±0.20^b^

Values are means ± SEM; n = 9–10 rats per treatment group.

^a,b^Within a row, values that do not share the same superscript letters are significantly (*P*<0.05) different from each other.

### Serum total-, HDL-cholesterol, and triglycerides

The effects of Orx and supplemental doses of vitamin E and/or aspirin on serum lipid parameters are presented in Table 2. Orchidectomy did not alter the levels of serum TG and HDL but significantly increased serum TC by 48% compared to Sham. Although the high-dose vitamin E exerts an intermediate effect in reducing TC, aspirin alone and combination of aspirin and vitamin E were more effective in reducing serum TC levels to the level of similar to Sham animals.

**Table 2:** Effect of orchidectomy (Orx), vitamin E and aspirin on serum lipid parameters.

	Sham	Orx			
Treatment (per kg diet)	Control	Control	500 IU Vit E	500 mg Aspirin	500 IU Vit E + 500 mg Aspirin
Total Cholesterol (mg/dL)	138±12.9^b^	205±12.2^a^	172±12.2^a^^b^	165±9.6^b^	164±13.8^b^
HDL-Cholesterol (mg/dL)	46.0±4.1	63.7±3.9	56.6±3.9	55.9±3.3	55.6±5.5
Triglycerides (mg/dL)	65.1±5.8	83.6±5.5	70.1±5.5	69.3±5.3	65.8±4.6

Values are means ± SEM; n = 9–10 rats per treatment group.

^a,b^Within a row, values that do not share the same superscript letters are significantly (*P*<0.05) different from each other.

### Atherosclerotic lesions, aortic fatty streak area, and progression of atherosclerosis lesions

**Fig 1:**
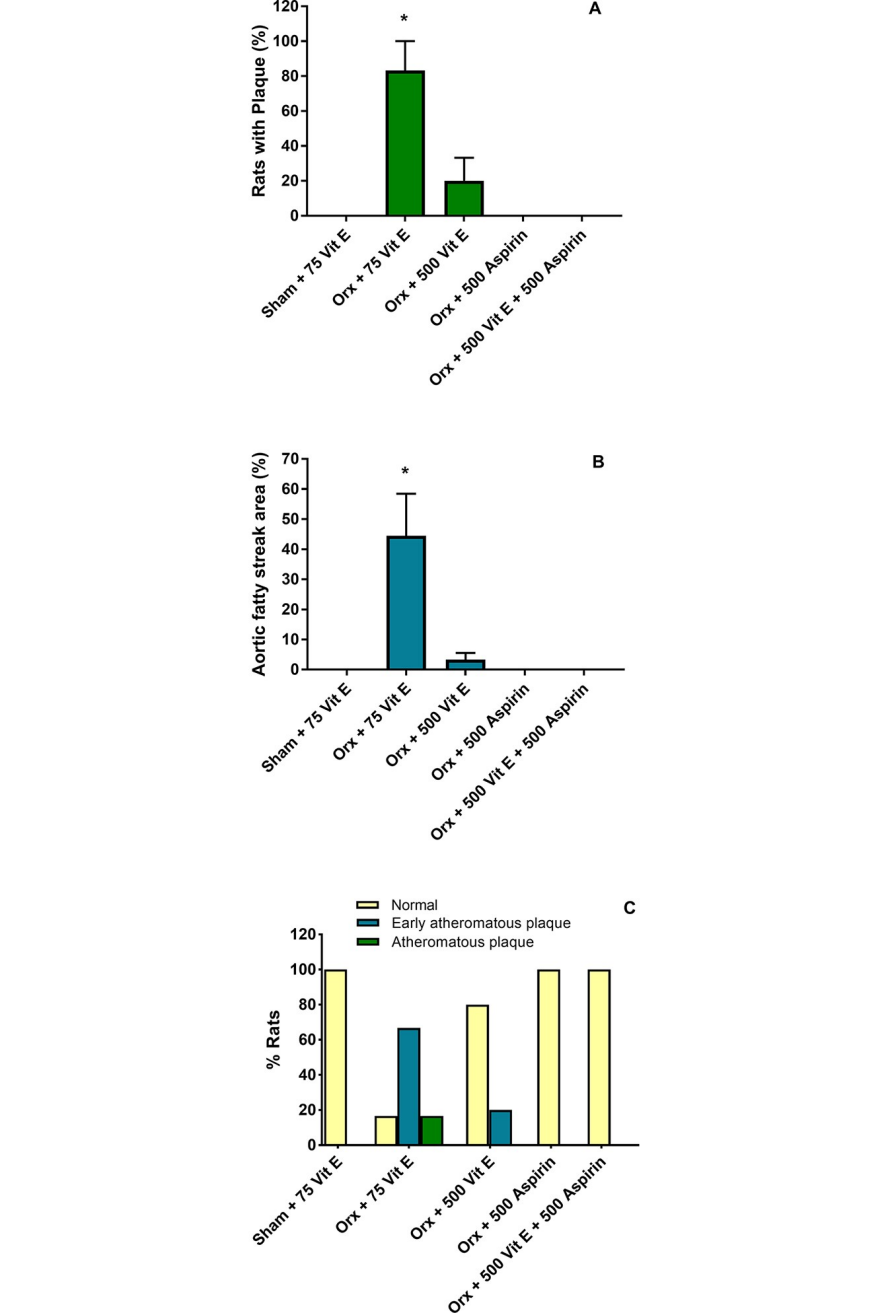
Effects of orchidectomy (Orx), vitamin E and aspirin on number of rats with atherosclerotic plaques (1A), aortic fatty streak area (1B), and progression of atherosclerotic lesions (1C). All these values (Figures A-C) are expressed as a percentage. Bars (1A and 1B) represent the percent means ± SEM from 9–10 rats per treatment group. *Asterisk denotes significant differences in comparison with other groups (*P*<0.05). Bars (1C) represent the distribution of rats (percentage) in the different degrees of lesion formation, n = 9–10 per group. The degree of lesion formation was classified as normal (without lesion formation), early atheromatous, and atheromatous.

At the end of the study, Sham animals had no atherosclerotic lesions, but approximately 83% of Orx rats developed atherosclerotic plaques (Fig 1A and Table 3). This percent was significantly reduced to less than 20% in Orx rats that received the supplemental dose of 500 IU vitamin E. In addition, 500 mg aspirin alone completely made the atherosclerosis plaques to disappear. Similarly, rats that received 500 IU vitamin E had diminished the expanded aortic fatty streak area (Fig 1B and Table 3) while 500 mg aspirin completely prevented and/or reversed the fatty streak area. In terms of atherosclerotic lesion progression (Fig 1C), the lesions in the Orx rats were of more advanced stages and 500 IU vitamin E inhibited the progression of atherosclerotic lesion. Orx rats that received 500 mg aspirin was completely normal similar to those of Sham animals.

**Table 3:** Effect of orchidectomy (Orx), vitamin E and aspirin on atherosclerotic lesions and aortic fatty streak area.

	Sham	Orx			
Treatment (per kg diet)	Control	Control	500 IU Vit E	500 mg Aspirin	500 IU Vit E + 500 mg Aspirin
Rats with plaque (%)	0.0±0.0^b^	83.3±16.7^a^	20.0±13.3^b^	0.0±0.0^b^	0.0±0.0^b^
Aortic fatty streak area (%)	0.0±0.0^b^	44.4±14.0^a^	3.3±2.2^b^	0.0±0.0^b^	0.0±0.0^b^

Values are means ± SEM; n = 9–10 rats per treatment group.

^a,b^Within a row, values that do not share the same superscript letters are significantly (*P*<0.05) different from each other.

### Bone mineral density

The effects of Orx, vitamin E and aspirin on BMD, BMC, and BMA of whole body, 3^rd^, 4^th^, and 5^th^ lumbar vertebra and right femur are presented in Table 4. Mean BMD and BMC values of Orx animals were significantly different than Sham animals. By the end of treatment, the mean BMD values of vitamin E, aspirin, or combined of vitamin E and aspirin treated groups were not different than those of Orx controls. BMA were not affected by Orx, vitamin E or aspirin supplementation.

**Table 4:** Effects of orchidectomy (Orx), vitamin E and aspirin on bone mineral density (BMD), bone mineral content (BMC), and bone mineral area (BMA) of 3^rd^, 4^th^, and 5^th^ lumbar vertebra and right femur.

	Sham	Orx			
Treatment (per kg diet)	Control	Control	500 IU Vit E	500 mg Aspirin	500 IU Vit E + 500 mg Aspirin
BMD (g/cm^2^)					
Whole body	0.187±0.001^a^	0.177±0.002^b^	0.174±0.002^b^	0.173±0.002^b^	0.177±0.001^b^
3^rd^ lumbar	0.242±0.0045^a^	0.218±0.0043^b^	0.215±0.0043^b^	0.214±0.0045^b^	0.223±0.0045^b^
4^th^ lumbar	0.256±0.0040^a^	0.223±0.0038^b^	0.218±0.0038^b^	0.220±0.0040^b^	0.223±0.0040^b^
5^th^ lumbar	0.256±0.0043^a^	0.228±0.0041^b^	0.225±0.0041^b^	0.224±0.0043^b^	0.232±0.0043^b^
Right femur	0.277±0.0037^a^	0.249±0.0035^b^	0.242±0.0035^b^	0.243±0.0035^b^	0.252±0.0037^b^
BMC (g)					
Whole body	15.9±0.197^a^	14.6±0.198^b^	14.4±0.299^b^	14.6±0.360^b^	14.7±0.277^a^^b^
3^rd^ lumbar	0.185±0.0060^a^	0.160±0.0057^b^	0.156±0.0057^b^	0.153±0.0060^b^	0.155±0.0060^b^
4^th^ lumbar	0.203±0.0059^a^	0.173±0.0056^b^	0.163±0.0056^b^	0.168±0.0059^b^	0.173±0.0059^b^
5^th^ lumbar	0.206±0.0063^a^	0.173±0.006^b^	0.170±0.006^b^	0.167±0.0063^b^	0.175±0.0064^b^
Right femur	0.717±0.0170^a^	0.625±0.0161^b^	0.608±0.0161^b^	0.611±0.0161^b^	0.648±0.0170^b^
BMA (cm^2^)					
Whole body	84.8±0.965	82.3±1.258	82.8±1.331	84.1±1.671	84.2±1.417
3^rd^ lumbar	0.765±0.0166	0.733±0.0163	0.722±0.0163	0.715±0.0172	0.706±0.0166
4^th^ lumbar	0.794±0.0167	0.778±0.0158	0.746±0.0158	0.760±0.0167	0.774±0.0167
5^th^ lumbar	0.807±0.0178	0.759±0.0169	0.754±0.0169	0.743±0.0178	0.752±0.0178
Right femur	2.58±0.0444	2.51±0.0421	2.51±0.0421	2.51±0.0178	2.57±0.0444

Values are means ± SEM; n = 9–10 rats per treatment group.

^a,b^Within a row, values that do not share the same superscript letters are significantly (*P*<0.05) different from each other.

### Trabecular microarchitectural of distal femur

The effects of Orx, vitamin E and aspirin on trabecular microarchitectural of distal femur are presented in Table 5. Analysis of data indicated that Orx significantly decreased trabecular bone volume (BV/TV), trabecular number (Tb.N), and connectivity density (Conn.D) when compared to the Sham animals. Neither the vitamin E nor aspirin were able to restore BV/TV, Tb.N and Conn.D in these Orx rats. Orchidectomy did not alter trabecular thickness (Tb.Th) and trabecular separation (Tb.Sp). However, 500 IU vitamin E, 500 mg aspirin, and combined of vitamin E and aspirin significantly lowered Tb.Th compared to Sham animals. In addition, 500 mg aspirin significantly increased Tb.Sp in comparison with Sham animals. Structure model index (SMI) were not affected by Orx, vitamin E or aspirin supplementation.

**Table 5:** Effects of orchidectomy (Orx), vitamin E and aspirin on trabecular microarchitectural parameters of distal femur.

	Sham	Orx			
Treatment (per kg diet)	Control	Control	500 IU Vit E	500 mg Aspirin	500 IU Vit E + 500 mg Aspirin
BV/TV (1)	0.137±0.013^a^	0.061±0.008^b^	0.054±0.008^b^	0.048±0.002^b^	0.055±0.007^b^
Tb.N (1/mm)	2.095±0.151^a^	1.293±0.151^b^	1.438±0.151^b^	1.136±0.110^b^	1.396±0.164^b^
Tb.Th (mm)	0.085±0.002^a^	0.075±0.002^a^^b^	0.071±0.002^b^	0.068±0.005^b^	0.070±0.003^b^
Tb.Sp (mm)	0.521±0.069^b^	0.793±0.069^a^^b^	0.737±0.069^a^^b^	0.930±0.087^a^	0.770±0.083^a^^b^
Conn.D (1/mm^3^)	26.63±2.03^a^	14.15±2.03^b^	13.99±2.03^b^	13.89±0.73^b^	15.77±1.04^b^
SMI	1.668±0.126	1.903±0.126	2.141±0.126	1.678±0.148	1.890±0.134

Values are means ± SEM; n = 6 rats per treatment group.

^a,b^Within a row, values that do not share the same superscript letters are significantly (*P*<0.05) different from each other.

### Urinary deoxypyridinoline (Dpd) and serum osteocalcin (OC)

The effects of Orx, vitamin E and aspirin on urinary Dpd and serum OC are presented in Table 6. No differences in serum levels of OC were noted among all treatment groups. Similarly, urinary Dpd were not affected by Orx, vitamin E or aspirin supplementation.

**Table 6:** Effects of orchidectomy (Orx), vitamin E and aspirin on biomarkers of bone metabolism.

	Sham	Orx			
Treatment (per kg diet)	Control	Control	500 IU Vit E	500 mg Aspirin	500 IU Vit E + 500 mg Aspirin
Urinary Dpd (nmol/mmol creatinine)	22.8±5.68	38.3±5.39	32.3±5.39	31.2±5.39	33.7±5.68
Serum Osteocalcin (ng/mL)	13.0±1.48	17.9±1.41	16.0±1.41	17.7±1.41	14.1±1.48

Values are means ± SEM; n = 9–10 rats per treatment group.

## Discussion

In an earlier study [15], we investigated the dose-dependent effects of vitamin E on aortic fatty lesion formation in Orx rats. In that study we found that 500 IU vitamin E significantly reduced formation of atherosclerotic lesions and aortic fatty streak areas compared to Orx control. This positive effect could be due, in part, to the mild effect of vitamin E on inflammatory markers due to its anti-oxidative properties. Low dose aspirin is also commonly prescribed for prophylactic purposes, in part, due to its anti-inflammatory properties. The present study assessed the extent to which vitamin E and aspirin individually or in combination prevent and/or reverse bone loss and atherosclerotic lesion formation in Orx rats.

TC and an elevated lipid profile all contribute to the development of atherosclerosis [41, 42]. Orchidectomy significantly elevated mean serum TC level compared to Sham. This increased TC level may be partially due to higher HDL concentration, as its concentration increased in comparison with the Sham rats, but the increase was not statistically significant. However, the Orx rats which received 500 IU supplemental dose of vitamin E had somewhat lower serum TC. Aspirin alone, and the combination of aspirin and vitamin E, were more effective in reducing serum TC levels to a level that was similar to Sham animals. Several studies by other investigators have demonstrated similar findings using an Orx rabbit model of atherosclerosis [43] and castration rat model [44]. Additionally, an epidemiological study [45] has reported that HDL levels in older adults are higher than in middle-aged men and this rise in HDL has been linked to decreased serum testosterone concentrations [46]. Nonetheless, Orx rats developed atherosclerotic lesions despite having higher HDL, suggesting that gonadal hormone deficiency may negatively contribute to the development of atherosclerosis independent of cholesterol status.

The present findings suggest that 500 IU vitamin E was able to reduce the development of atherosclerotic lesion formation and aortic fatty streak area compared to Orx control. More importantly, 500 mg aspirin alone completely reversed the fatty streak area and made the atherosclerotic lesions disappear. The anti-atherogenic effects of aspirin are partially due to its anti-platelet effects which leads to a decrease in clot formation, thus a decrease in arterial occlusion [47]. Moreover, the creation of fatty lesions or plaques are dependent up on the solubility of the cholesterol [48]. Higher membrane cholesterol concentrations have been found to result in increased plaque formation, while increasing the aspirin concentration in those membranes serves to dissolve the plaques [48]. This is an interesting finding because human observational studies have been inconclusive regarding the beneficial effects of regular aspirin usage [49]; safety concerns regarding other aspects of supplementation such as increased risk of hemorrhagic stroke [50, 51] or the effects of aspirin on various organs are also reasonable. Though vitamin E supplementation does moderately improve lipid parameters [52], the combination of aspirin and vitamin E would likely serve to be the most effective in reducing heart disease risk and preventing the development of fatty lesions in human populations.

Our findings of the present study also suggest that biomarkers of bone formation and resorption, i.e. urinary Dpd and serum OC, were not affected by vitamin E or aspirin supplementation. In terms of BMD of whole body, lumbar vertebrae and femurs, our data showed that aspirin supplementation did not reverse or alter the Orx-induced bone loss in comparison with Orx controls. Aspirin did not reverse age and hormone deficiency related decreases in BMD, and instead appears to have a negative effect on bone health. Due to the inflammatory nature of the development of osteoporosis, this finding is interesting; one would think that decreases in the inflammatory process would lead to decreased bone turnover and possibly bone formation. Though aspirin works to inhibit pathways that stimulate osteoclasts, the present study suggests that aspirin supplementation may in fact increase fracture risk as shown by increased in trabecular separation. Based on the result of previous studies, aspirin have been shown to moderately improve BMD and bone structural in young rats models of osteoporosis [27, 29, 30]. So far there is inconclusive evidence on humans. Epidemiological studies have shown that aspirin does not appear to improve fracture risk [53, 54], but may in fact increase fracture risk [55], which supports our findings. Fracture risk is dependent upon bone strength, which includes multiple factors but specifically microarchitecture [56]. The ability of trabecular structure to dissipate pressure brought about by normal activity, exercise, or accidental falls changes based on trabecular architecture; the increase in trabecular separation in the aspirin group suggests a decreased ability to handle greater pressure, thus an increase in fracture risk.

The reason behind this increased risk may have to do with the aspirin dosage. The given dose in this study is comparable the highest dose that is safe to consume in humans. The amount of aspirin consumed by rats was approximately the equivalent of the maximum safe dose of 1000 mg/70 kg human, as the rats consumed about 16g of diet per day. Because aspirin works to inhibit inflammation via COX pathways, COX-2 knockout mice have been shown to have significantly decreased BMD, indicating an integral role for inflammation in the bone remodelling process [57]. While too much inflammation may also cause excessive bone resorption, it would appear that too little inflammation results in a similar effect. Unfortunately, the present study did not specifically measure inflammatory biomarkers. Future studies are needed to assess inflammatory status in order to further understand this relation.

Our previous studies [15, 40, 58–61] have shown that vitamin E has multiple health benefits, but our findings in this study regarding its bone protective effects proved unexpected. Knowing that vitamin E is a potent antioxidant, which works to relieve oxidative stress, and thusly inflammation, we find that vitamin E did not improve BMD and BMC in male rat model of osteoporosis even though osteoporosis is linked to inflammation. Ahmad et al. [62] previously demonstrated that vitamin E reduced the oxygen-derived free radical stimulation of osteoclastic bone resorption which would theoretically lead to an increase in BMD, and previous studies [58–61] by our lab have shown that vitamin E is protective against bone loss. However, because osteoporosis is not contingent upon BMD alone, vitamin E may still prove to be helpful in improving other markers related to bone health. We have shown that a high dose of vitamin E (500 mg) in hindlimb unloaded male rats improved trabecular bone microarchitecture [60]; this is important for bone integrity and can help prevent fractures even if BMD does not significantly increase. COX-2 levels were also decreased in this model at high levels of vitamin E supplementation [60]. Another study by our lab showed that supplementation of vitamin E at levels of 300 mg or more decreases osteoclastogenesis as well as the cells that support osteoclast differentiation [59].

While our previous evidence indicates that positive changes do occur regarding bone health, this study did not yield such results. However, while the aspirin only proved detrimental for bone health, the combination therapy (vitamin E and aspirin) prevented the increase in trabecular separation. Based on this data, a combination therapy approach may prove the most useful in preventing both fractures and atherosclerotic lesion development.

Though vitamin E supplementation was able to reduce the development of atherosclerotic lesion formation and aortic fatty streak area compared to Orx control, aspirin was the most effective intervention. However, because aspirin was also shown to negatively alter bone microarchitectural, the combination of aspirin and vitamin E would likely serve to be the most effective and healthful regimen for individuals looking to decrease heart disease risk while also preserving bone quality. This study also indicates that while using a high dosage of aspirin to prevent a cardiovascular event is technically safe, it may inadvertently be causing a loss of bone quality. Further research using different aspirin dosages to investigate its effect on bone quality and aortic lesions is still needed.
